# Long‐Term Efficacy and Resting‐State Functional Magnetic Resonance Imaging Changes of Deep Brain Stimulation in the Lateral Habenula Nucleus for Treatment‐Resistant Bipolar Disorder

**DOI:** 10.1002/brb3.70899

**Published:** 2025-09-30

**Authors:** Chao Jiang, Peijing Du, Lingxiao Guan, Chunhua Hu, Zhiyan Wang, Tengteng Fan, Jian Wang, Zhiqiang Cui

**Affiliations:** ^1^ College of Life and Health Sciences Institute of Neuroscience Northeastern University Shenyang China; ^2^ Department of Neurosurgery Chinese People's Liberation Army of China General Hospital Beijing China; ^3^ CAS Key Laboratory of Mental Health Institute of Psychology Chinese Academy of Sciences Beijing China; ^4^ National Engineering Research Center of Neuromodulation School of Aerospace Engineering Tsinghua University Beijing China; ^5^ Department of Psychology University of Chinese Academy of Sciences Beijing China; ^6^ Peking University Sixth Hospital Peking University Institute of Mental Health NHC Key Laboratory of Mental Health (Peking University) Beijing China; ^7^ National Clinical Research Center for Mental Disorders (Peking University Sixth Hospital) Peking University Beijing China

**Keywords:** deep brain stimulation, fractional amplitude of low‐frequency fluctuations, lateral habenula nucleus, resting‐state fMRI, resting‐state functional connectivity, treatment‐resistant bipolar disorder

## Abstract

**Background:**

To explore the long‐term efficacy and resting‐state functional magnetic resonance imaging (fMRI) changes of lateral habenula nucleus (LHb) deep brain stimulation (DBS; LHb‐DBS) for treatment‐resistant bipolar disorder (TRBD).

**Methods:**

An 18‐year‐old woman with TRBD received bilateral LHb‐DBS. We assessed changes in Hamilton Depression Scale‐17 (HDRS‐17), Bech‐Rafaelsen Melancholia Scale (BRMS), Hamilton Anxiety Scale (HAMA), and Pittsburgh Sleep Quality Scale (PSQI) scores from preoperative baseline to postoperative continuous 24‐month follow‐up. Brain activity and resting‐state functional connectivity (rsFC) were examined off‐stimulation at 0.6 and 15 months post‐LHb‐DBS. Overall improvement and adverse events were analyzed.

**Results:**

Continuous 24‐month follow‐up showed average improvements from baseline of 65.33%, 54.90%, 63.33%, and 48.72% for HDRS‐17, BRMS, HAMA, and PSQI scores, respectively. At the final follow‐up, improvement was 96.00%, 88.24%, 84.85%, and 69.23%, respectively. Resting‐state fMRI results revealed an increase in fractional amplitude of low‐frequency fluctuations (fALFF) within the putamen, ventral tegmental area (VTA), and substantia nigra pars compacta (SNc) over 15 months of continuous bilateral LHb stimulation when DBS was off. From baseline to 15 months, fALFF in the putamen, VTA, and SNc increased by 1.68%, 6.36%, and 1.10%, respectively. Consistently reduction in rsFC was observed between the left nucleus accumbens (NAcc) and left hippocampus. Over the 15 months of continuous stimulation, rsFC decreased by 72% from baseline.

**Conclusions:**

Long‐term LHb‐DBS can control symptoms and improve the quality of life in patients with TRBD. This may be attributed to an increase in fALFF in the putamen, VTA, and SNc, and a reduction in rsFC between the left NAcc and left hippocampus.

## Graphical abstract

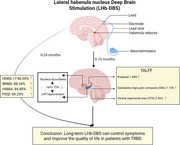

## 1 Introduction

Bipolar disorder (BD) is a multifaceted mental health condition characterized by the cyclical occurrence of depressive, manic, and mixed episodes, which can be stratified into BD I, BD II, BD predominantly mixed‐episode presentations, or BD with psychotic features. Notably, depression often constitutes the foremost manifestation of the BD (Nierenberg et al. 2023; Tondo et al. 2017). Pharmacotherapy (Sachs et al. 2011) and psychotherapy (Swartz and Swanson 2014) serve as primary treatment modalities for BD. Treatment resistance in bipolar disorder (TRBD) lacks a universally standardized definition but is often clinically defined by a failure to achieve sustained remission despite adequate trials of at least two evidence‐based pharmacological classes, including mood stabilizers (e.g., lithium and valproate) and second‐generation antipsychotics, often in combination with psychotherapy (Sachs 1996; Fountoulakis et al. 2017). This definition aligns with the rationale for considering advanced interventions, such as deep brain stimulation (DBS), in severe, refractory cases. Recently, there has been a shift toward exploring advanced brain stimulation techniques for the treatment of BD, such as electroconvulsive therapy, repetitive transcranial magnetic stimulation (rTMS), transcranial direct current stimulation, magnetic seizure therapy, vagus nerve stimulation, and DBS (Mutz 2023). DBS has become an established treatment for patients with a wide variety of conditions, which include movement disorders, psychiatric disorders, epilepsy, and pain (Schulder et al. 2023). However, despite its growing recognition, there is limited empirical evidence delineating target sites for the application of DBS for BD treatment. The most prominent targets are the subcallosal cingulate (SCC) (Cha et al. 2024), ventral capsule/ventral striatum (VC/VS) (Malone et al. 2009), superolateral branch of the medial forebrain bundle (Schlaepfer et al. 2013), ventral anterior limb of the internal capsule (vALIC) (Graat et al. 2020), and the lateral habenula nucleus (LHb) (C. Zhang et al. 2022). The safety profile and therapeutic efficacy of such DBS targets in BD remain subjects of ongoing inquiry and debate.

Recent studies have shown that the LHb plays a key role in the etiology of depression, inhibition of the LHb relieves depressive symptoms (Yang et al. 2018), and LHb‐DBS relieves symptoms in patients with TRBD (C. Zhang et al. 2022). These findings offer a clinical precedent for the application of LHb‐DBS to TRBD management. The LHb has emerged as a novel target for ameliorating neuropsychiatric symptoms; however, the precise role of the LHb in treating refractory neuropsychiatric conditions requires further investigation (Abraham et al. 2023).

The LHb is located in the dorsal thalamus and is an important link between the forebrain and brain stem monoaminergic nuclei, moreover, it is the source of the negative reward signal of dopamine neurons (Matsumoto and Hikosaka 2007). The LHb plays a key role in encoding negative reward and behavioral motivation (Hu et al. 2020). Dysregulation in reward processing is crucial in the pathophysiology of BD (Berk et al. 2007), and neural abnormalities in reward circuits are associated with BD vulnerability (Nusslock and Alloy 2017). We used resting‐state functional magnetic resonance imaging (fMRI) of the reward circuit in patients with TRBD undergoing LHb‐DBS. We focused on the basal ganglia, which includes the putamen, nucleus accumbens (NAcc), and ventral tegmental area (VTA), all of which are crucial for reward processing (Schultz 2016). Research has shown alterations in the shape (Hwang et al. 2006), size (Strakowski et al. 2002), and function (Strakowski et al. 2005) of the basal ganglia in patients with BD. The putamen, which is vital for motor control and reward processing, exhibits lower activation in BD patients during manic states than controls (Chen et al. 2011). The VTA and substantia nigra pars compacta (SNc), rich in dopaminergic neurons, are essential for reward processing and reinforcement learning. DBS targeting the striatum impacts the VTA and SNc owing to their interconnectedness within the reward circuit (Stamatakis et al. 2013; Hikosaka 2010). Although definitive evidence of disrupted connectivity between the NAcc and hippocampus during manic episodes is lacking, research has suggested that patients with BD have aberrant functional coupling between the NAcc and various cerebral loci. This disrupted connectivity may be related to manic symptoms. Indeed, it has been reported that increased structural connectivity of the NAcc, correlates with a propensity for hypomania (Damme et al. 2017).

In this paper, we report the long‐term efficacy and resting‐state fMRI changes in a patient with TRBD who underwent LHb‐DBS.

## 2 Methods

### 2.1 Patient

**FIGURE 1:**
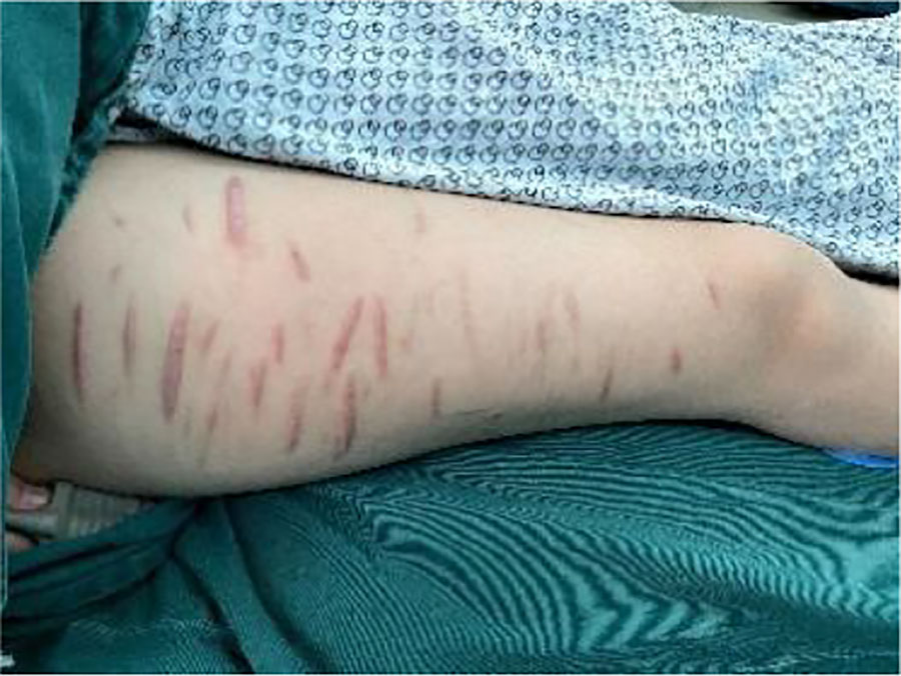
Photograph of scars on the patient's right thigh resulting from prior self‐injurious behavior. During physical examination, scars were found to have different lengths of 3–5 cm, which were caused when the patient was self‐disabled.

This case report details the long‐term outcomes of a single patient with severe TRBD who underwent LHb‐DBS. The patient was an 18‐year‐old woman who had experienced persistent low mood for over 5 years, which had continuously worsened over the past 3 years. In April 2020, the patient experienced recurrent episodes of emotional agitation accompanied by self‐injurious behavior and received a dual diagnosis of major depressive disorder and BD. During subsequent follow‐ups, the patient consistently reported feelings of depression, tension, and anxiety, which resulted in episodes of uncontrollable weeping and self‐harm (Figure 1). She had been treated with a regimen of medications, which included psychotropic drugs such as venlafaxine hydrochloride, sodium valproate, and lorazepam, as well as psychological therapy and repetitive TMS. Despite these interventions, the therapeutic response was poor, and the patient was unable to resume her normal academic pursuits. In light of these challenges, the patient sought further treatment and was admitted to our hospital for inpatient care. Following comprehensive evaluations by two psychiatrists, the patient was definitively diagnosed with BD with a concurrent depressive episode. We conducted a series of psychometric assessments and the patients scored 25 on the Hamilton Depression Rating Scale (HDRS), 33 on the Hamilton Anxiety Scale (HAMA), and 25 on Bech‐Rafaelsen Melancholia Scale (BRMS). A detailed physical examination and MRI scan were performed according to inclusion and exclusion criteria, and other psychiatric diagnoses were ruled out in accordance with the Diagnostic and Statistical Manual, Fifth Edition (DSM‐5) (Svenaeus 2014). Upon admission, the patient was on the following medications: magnesium valproate sustained‐release tablets 1.5 g/day, tandospirone citrate 30 mg/day, and other Chinese patent medicines. The patient and her family provided informed consent to participate in the clinical trial, which was approved by the Ethics Committee of the Chinese People's Liberation Army General Hospital (Chinese Clinical Trials 2100045363).

### 2.2 Target Coordinates, Surgical Path, and Surgical Method

We utilized the Leksell stereotactic planning system (Elekta, Stockholm, Sweden) to fuse the T1‐ and T2‐weighted images, to design the target and needle track, and obtain the target coordinates left: *x* = 2, *y* = −12, *z* = 2; right: *x* = 2, *y* = −13, *z* = 0. To ensure the safety of implantation, we avoid the caudate nucleus, lateral ventricle, precentral gyrus, pyramidal tract, and cortical and thalamic vessels. The surgical path design and detailed surgical methods have been reported previously (Cui et al. 2023).

### 2.3 Programming Control, Efficacy Evaluation, and Follow‐Up

**FIGURE 2:**
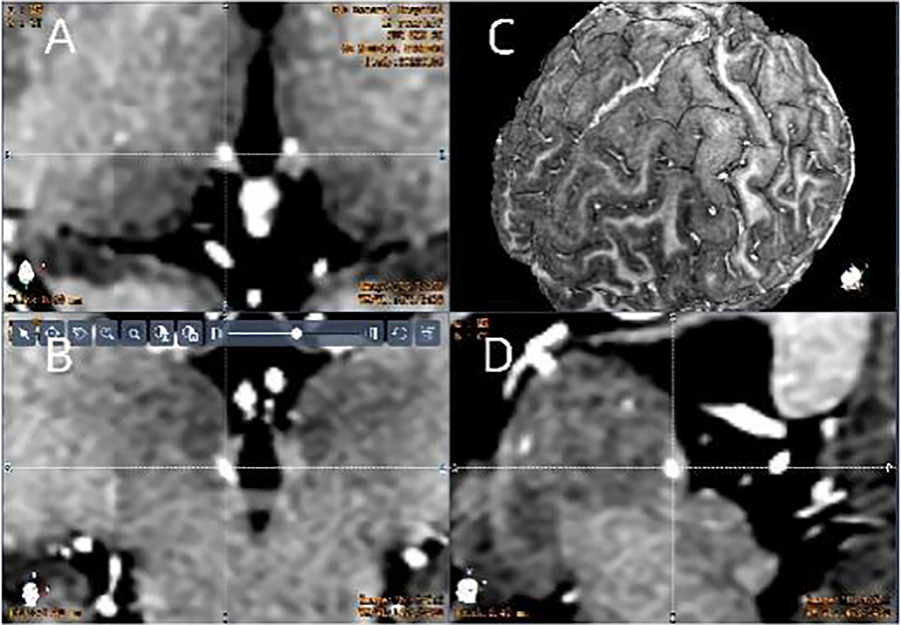
Achieved electrode positions in the lateral habenula nucleus (LHb). Fusion of postoperative CT (electrode artifacts white dots) with preoperative functional MRI. The Hb is visible (with the intersection of the white dotted line indicating the center of the left Hb) in 3D T1‐weighted MR images. (A) Axial; (B) coronal; (C) whole‐brain imaging; (D) sagittal.

The patient underwent outpatient programming control 1 month after surgery, head computed tomography was reviewed before startup, and the fused images were compared with preoperative MRI images to determine the accuracy of the electrode position (Figure 2). Stimulation parameters were based on the unipolar test results, and the patient themselves feedback themselves. The pulse width was 90 µs, the frequency was 160 Hz, and the bilateral voltage was 1.5–3.0 V. The subsequent parameters were optimized according to the patient's symptoms (Table 1). We used the following scales to assess postoperative symptoms: the HDRS‐17 (Hamilton 1960) (symptom improvement criterion: 25%–49% improvement = improvement; 50% improvement = response; a score of 8 = remission), BRMS (Bech 2002), HAMA (Hamilton 1959), Montgomery Depression Scale (MADRS) (Montgomery and Asberg 1979), Quick Inventory of Depressive Symptomatology‐Self‐Rated (QIDS‐SR) (Rush et al. 2003), and Pittsburgh Sleep Quality Scale (PSQI) (Buysse et al. 1989). The improvement rate of the above scales was measured using to the following formula: improvement rate = (preoperative score − postoperative score) / preoperative score × 100%.

**TABLE 1:** The scores and improvement rates on various scales between preoperative and postoperative 0–24 months of the patient (HDRS‐17; MADRS; QIDS‐SR; HAMA; PSQI; BRMS).

	Preoperative	Postoperative scores (improvement rate)(%)									Average improvement rate (%)
	Baseline	1 m	3 m	6 m	9 m	12 m	15 m	18 m	21 m	24 m	
HDRS‐17	25	23 (8.00)	10 (60.00)	14 (44.00)	3 (88.00)	3 (88.00)	18 (28.00)	1 (96.00)	5 (80.00)	1 (96.00)	65.33%
MADRS	34	32 (5.88)	18 (47.06)	21 (38.24)	6 (82.35)	6 (82.35)	9 (73.53)	8 (76.47)	2 (94.12)	2 (94.12)	66.01%
QIDS‐SR	19	18 (5.26)	12 (36.84)	16 (15.79)	12 (36.84)	5 (73.68)	18 (5.26)	6 (68.42)	7 (63.16)	4 (78.95)	36.02%
HAMA	33	29 (12.12)	20 (39.39)	23 (30.30)	11 (66.67)	6 (81.82)	9 (72.73)	4 (87.88)	2 (93.94)	5 (84.85)	63.30%
PSQI	13	6 (53.85)	6 (53.85)	9 (30.77)	6 (53.85)	2 (84.62)	6 (53.85)	6 (53.85)	8 (38.46)	4 (69.23)	48.72%
BRMS	34	24 (29.41)	23 (32.35)	26 (23.53)	20 (41.18)	18 (47.06)	12 (64.71)	11 (67.65)	0 (100.00)	4 (88.24)	54.90%

*Note*: Preoperative baseline: the scores on various scales (HDRS‐17, MADRS, QIDS‐SR, HAMA, PSQI, BRMS) before surgery of the patient. Postoperative scores: the scores on various scales (HDRS‐17, MADRS, QIDS‐SR, HAMA, PSQI, BRMS) after surgery of the patient during 1, 3, 6, 9, 12, 15, 18, 21, and 24 months follow‐up period. Improvement rates: improvement rate of various scales during 1, 3, 6, 9, 12, 15, 18, 21, and 24 months follow‐up period in postoperative scores compared to baseline. Average improvement rate: average improvement rates of various scales during 1, 3, 6, 9, 12, 15, 18, 21, and 24 months follow‐up period compared to preoperative baseline.

### 2.4 MRI Acquisition

Similar to our previous reported, during the DBS shutdown state both preoperative baseline and postoperative structural and fMRI images at 0.6 and 15‐month follow‐ups were acquired on a 3.0 Tesla Prisma (Siemens, Erlangen, Germany) MRI scanner equipped with a 64‐channel head coil. The patient underwent a preoperative baseline scan with structural MRI and six resting‐state fMRI runs, and postoperative scans with structural MRI, three fMRI runs without stimulation, and three fMRI runs with stimulation, respectively. There was a 30‐min wash‐out period before each DBS‐off acquisition. Structural images were acquired using a sagittal magnetization‐prepared rapid gradient echo T1‐weighted sequence (0.7 mm isotropic resolution, field of view [FOV] = 224, FOV phase = 100%, slice thickness = 0.7 mm, repetition time [TR] = 2200 ms, echo time [TE] = 2.48 ms, and flip angle = 8°). Functional images were acquired using an echo‐planar imaging sequence (voxel size = 3.0 × 3.0 × 3.0 mm, FOV = 216, FOV phase = 100%, slice thickness = 3.0 mm, TR = 3000 ms, TE = 30 ms, and flip angle = 85°).

#### 2.4.1 Resting‐State fMRI Data Preprocessing

We conducted preprocessing of the fMRI data using the CONN toolbox (22.a) (RRID: SCR_009550) (Whitfield‐Gabrieli 2012) in conjunction with customized MATLAB R2021B (The Mathworks, Natick, MA, USA) scripts. The preprocessing pipeline comprised slice timing correction, head motion realignment, coregistration of structural and functional images, and spatial normalization to the standard Montreal Neurological Institute space with voxel size resampled to 3 × 3 × 3 mm^3^. Spatial smoothing was applied using a Gaussian kernel with a full‐width‐half‐maximum of 4 × 4 × 4mm^3^. Nuisance regression was performed to remove motion artifacts and physiological noise, which involved regression of the Friston 24 motion parameters and signals from white matter and cerebrospinal fluid (Friston et al. 1996), as well as the removal of linear trends.

#### 2.4.2 Fractional Amplitude of Low‐Frequency Fluctuations (fALFF) and Resting‐State Functional Connectivity (rsFC) Calculation

Regions of interest (ROIs) related to reward circuits were defined using the Anatomical Atlas Labeling version 3 (AAL3) atlas (https://www.oxcns.org/aal3.html), ROIs were bilateral medial and lateral orbital gyri, bilateral putamen, bilateral NAcc, VTA, SNc, and raphe nuclei. The fALFF of each ROI was computed by averaging voxel‐wise fALFF values within the region at baseline, Months 0, 6, and 15. The computation off ALFF values was conducted using the DPABI V7.0 230110 software (http://rfmri.org/dpabi) (Yan et al. 2016). The time series for each voxel was transformed using the Fast Fourier Transform to obtain the power spectrum followed by the calculation of the square root of the power spectrum at each frequency. The fALFF value for each voxel was derived by computing the ratio of the power spectrum within a predefined frequency band (i.e. 0.01–0.10 Hz) to that of the entire frequency range (0–0.167Hz).

The rsFC analysis was conducted using the DPABI V7.0 230110 software (http://rfmri.org/dpabi) (Yan et al. 2016). We extracted the time courses of each ROI by averaging the time courses of all voxels within each ROI. Pearson's correlation coefficients were computed between every pair of ROIs and subsequently converted into Z‐scores using Fisher's z transformation. Graphs were produced using BrainNet Viewer (Xia et al. 2013) and GraphPad PRISM 8.0 (GraphPad Software, Inc, CA).

## 3 Results

### 3.1 Postoperative Efficacy of LHb‐DBS

Following a comprehensive 24‐months postoperative follow‐up period, substantial improvement was observed across a wide range of psychiatric manifestations, including depression, anxiety, and mania. This improvement translated into a marked enhancement in the patient's individual's quality of life. The HDRS‐17 score showed a mean improvement of 65.33% from the baseline scores. Symptoms of anxiety decreased considerably, alongside concomitant elevations in both quality of life and sleep quality. The MADRS and QIDS‐SR scores showed mean improvement rates of 66.01% and 36.02%, respectively; at the final follow‐up, the improvements were 94.12% and 78.95%, respectively. Similarly, the HAMA score showed mean and final follow‐up improvement rates of 63.30% and 84.85%, respectively. The PSQI score showed a mean improvement of 48.72% and a final follow‐up improvement rate of 69.23% (Table 1).

The BRMS scores improvement on average by 54.90% over the 24‐month postoperative period, which culminated in a final follow‐up improvement rate of 88.24%. The patient's mother had recounted that during periods of acute distress, the patient would lock herself in her room, occasionally erupt into fits of rage accompanied by vehement verbal outbursts toward family members, and engage in self‐harm that left numerous scars measuring 3–5 cm on the patient's forearms and shins. However, after the 24‐month postoperative follow‐up, there had been a marked discernible reduction in manic and depressive episodes, with no further instances of self‐injurious behavior. The patient reported a greater sense of control over these emotional fluctuations and expressed satisfaction with the surgical outcome. Following the administration of LHb‐DBS therapy, the patient exhibited effective management of depressive symptoms, with progressive improvement in overall health status. This enabled the patient to reintegrate into academic life, which led to a successful transition into higher education following the national college entrance examination. The patient reported notable improvement in concentration and interpersonal interactions compared with those before the surgery. Importantly, the commencement of stimulation did not provoke any adverse sequelae commonly linked to neurosurgical interventions or long‐term stimulatory protocols. Apart from a fleeting episode of vertigo upon initial stimulation, which abated following subsequent to meticulous parameter adjustment, there were no complications during the perioperative period.

### 3.2 fMRI Outcomes

**FIGURE 3:**
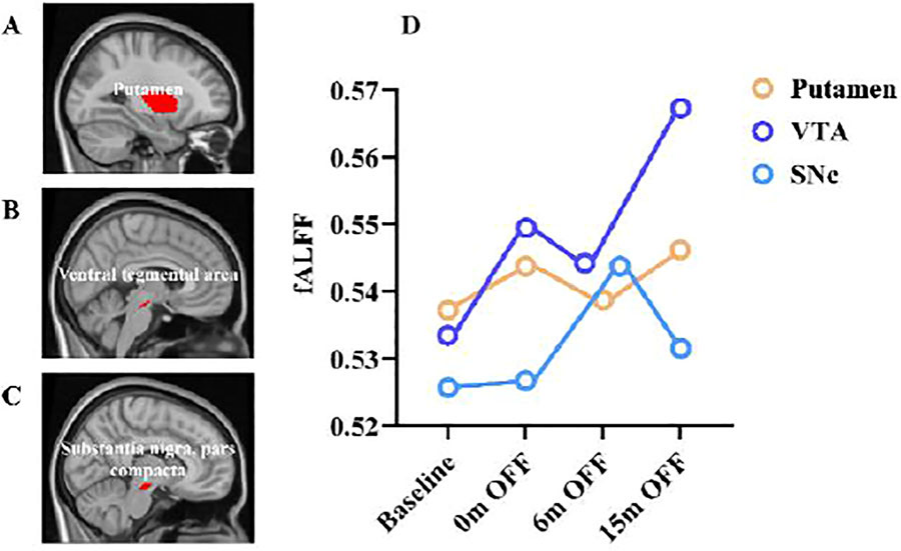
Chronic stimulation induced rsFC changes of the putamen, VTA, and SNc during baseline to 15 months. (A) The location of putamen; (B) the location of VTA; (C) the location of SNc; (D) individual data showed that the fALFF of putamen, VTA, and SNc increased at 15 months when DBS was off.

We examined fALFF within the predefined ROIs at baseline, Months 0, 6, and 12 when DBS was turned off. Although fluctuations were observed at various time points, we found an increase in fALFF in the putamen, VTA, and SNc over the 15 months of continuous bilateral LHb‐DBS (Figure 3). Specifically, fALFF in the putamen, VTA, and SNc increased by 1.68%, 6.36%, 1.10% from baseline, when DBS was turn off at month 15.

**FIGURE 4:**
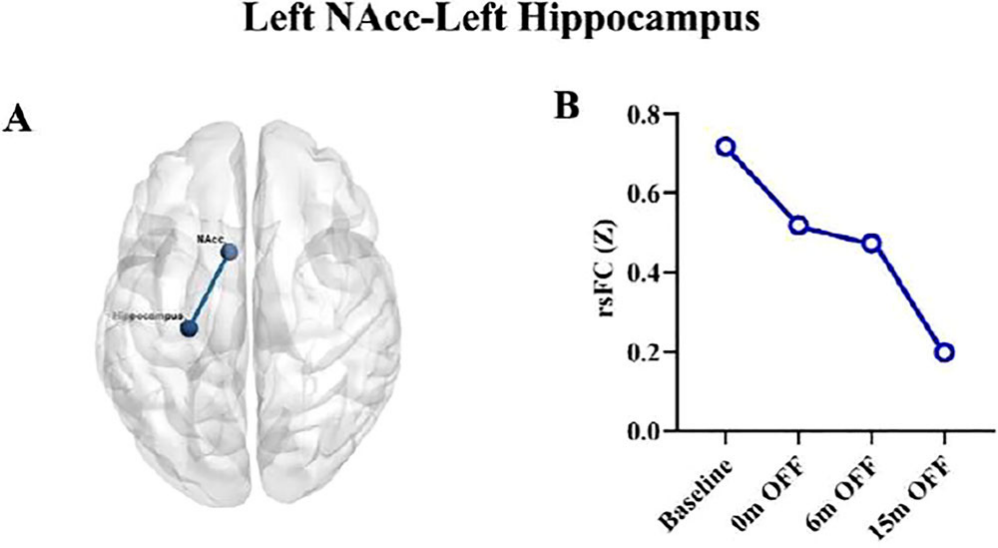
Chronic stimulation induced rsFC changes of the left NAcc‐left hippocampus during baseline to 15 months. (A) The rsFC of left NAcc‐left hippocampus; (B) individual data showed consistent decreases in the rsFC of left NAcc‐left hippocampus from baseline to 15 months when DBS was off.

A consistently reduction rsFC was observed between the left NAcc and left hippocampus from preoperative baseline to 15 months during which DBS was turned off at all time points (0 months, 6 months, and 15 months). Specifically, rsFC Z‐scores between the left NAcc and left hippocampus were 0.72 at baseline, 0.52 at Month 0, 0.47 at Month 6, and 0.12 at Month 15. The rsFC of left NAcc‐left hippocampus showed a 72% decrease from baseline to 15 months of continuous stimulation (Figure 4).

## 4 Discussion

The management of TRBD remains a significant clinical challenge, as conventional treatments often yield suboptimal outcomes in a subset of severely ill patients (McIntyre et al. 2020). In this case report, we observed that long‐term bilateral LHb‐DBS was associated with substantial and sustained improvements in depressive, anxious, and manic symptoms, as well as sleep quality, in a patient with severe TRBD. Furthermore, resting‐state fMRI revealed potential neuroplastic changes within the reward circuit accompanying these clinical benefits.

### 4.1 Depression Symptoms, Anxiety Symptoms, and Sleep Quality Following LHb‐DBS

In a pioneering study examining the utility of SCC‐DBS for treatment‐resistant depression (TRD), a cohort of unipolar depression (*n *= 10) and BD (*n *= 7) patients were subjected to an open‐label, chronic stimulation intervention protocol (monopolar stimulation, frequency = 130 Hz, pulse width = 91 µs, current = 6 mA) initiated 1 month postoperatively. A striking 50% reduction in HDRS‐17 scores was achieved for all 17 patients after 6 months of uninterrupted stimulation (Cha et al. 2024). Similarly, our patient exhibited a 44% decline in HDRS‐17 scores over the same period, which is consistent with the therapeutic gains reported previously. These findings substantiate the consistent effectiveness of SCC‐DBS in alleviating depressive symptoms in BD patients. It is worth noting that our initial approach involved synchronous bilateral stimulation (frequency = 160 Hz, pulse width = 90 µs).

Besides, revealed progressive reductions in HDRS‐17 scores at 24 weeks (*n* = 17), 1 year (*n* = 14), and 2 years (*n* = 12), averaging 43.6%, 43.0%, and 70.1% from baseline, respectively (Cha et al. 2024). In contrast, we observed recorded escalating improvement rates of HDRS‐17 score of 44.0% at 6 months, 88.0% at 12 months, and 96.0% at 24 months, which indicates superior long‐term efficacy of LHb‐DBS over SCC‐DBS for the treatment of BD. Another LHb‐DBS study targeting TRD that involved six BD patients and one TRD patient, yielded fluctuating HDRS‐17 improvement rates of 49% at 1 month (*n* = 7), 56% at 3 months (*n* = 5), 46% at 6 months (*n* = 5), and 64% at 12 months (*n* = 3) (C. Zhang et al. 2022). Our patient mirrored this pattern with HDRS‐17 score improvements rates of 8%, 60%, 44%, and 88% at corresponding intervals, which demonstrates similar amelioration of depressive symptoms.

Regarding anxiety symptoms, the aforementioned study noted enhancements of 49%, 61%, 48%, and 70% at 1, 3, 6, and 12 months, whereas our patients demonstrated more modest short‐term gains of 12.12%, 39.39%, and 30.30% at 1, 3, and 6 months, respectively (C. Zhang et al. 2022). Nonetheless, long‐term improvement rates surged to 81.82% at 12 months, which surpassed the findings of previous reports. This suggests that LHb‐DBS offers delayed yet favorable outcomes for BD‐related anxiety.

In terms of sleep quality, previous studies exhibited improvement rates of 42% versus 53.85%, 30% versus 30.77%, 28% versus 53.85%, and 28% versus 84.62% at 1, 3, 6, and 12 months, respectively (C. Zhang et al. 2022). Our cohort marginally outperformed the first study in terms of overall improvement in sleep quality. However, the limited sample size did not allow statistical validation, and individual variability must be considered. Nevertheless, our observations support current evidence endorsing LHb‐DBS as a modality for treating depression, anxiety, and sleep disturbances in patients with BD.

Zhang et al. reported in their case study on LHb‐DBS in a BD patient that high‐frequency stimulation (3 V, 120 µs, 130 Hz) effectively alleviated depressive symptoms. Furthermore, subsequent adjustment to low‐frequency stimulation (2 V, 60 µs, 60 Hz) 3 months later led to better control of depression and concurrent improvement in sleep quality (C. Zhang et al. 2019). These findings suggest that low‐frequency Hb‐DBS may offer comparable therapeutic benefits for patients with BD. However, without explicit reporting of improvement rates, it is not possible to perform comparative analyses. Future investigations with larger samples are needed to rigorously compare the efficacy of low‐ and high‐frequency LHb‐DBS protocols for treating BD.

### 4.2 Mania Symptoms Following LHb‐DBS

The patient displayed marked improvement in manic symptoms. This improvement was characterized by increased emotional equilibrium and a pronounced reduction in the irritability and the incidence of manic episodes. This was accompanied by enhanced emotional self‐regulation, circumventing the symptom intensification frequently observed upon DBS initiation. Torres et al. (2017) demonstrated the successful amelioration of depressive symptoms in BD‐I patients using CG24/25‐DBS without exacerbating hypomania symptoms. Furthermore, three additional studies, involving a total of nine BD‐II patients, reported that DBS efficiently curtailed depressive symptoms, and only one patient exhibited the emergence of hypomania. Upon cessation of stimulation, the hypomanic symptoms abated entirely, which demonstrated that DBS is a safe and efficacious treatment for BD that does not exacerbate hypomanic symptoms (Malone et al. 2009; Schlaepfer et al. 2013; Holtzheimer et al. 2012).

In a vALIC‐DBS study (*n* = 5), four patients experienced transient hypomanic exacerbations, which resolved after the adjustment of stimulation parameters and pharmacotherapy. Notably, no significant alterations in Young Mania Rating Scale scores were observed (Graat et al. 2020). In a study investigating the application of LHb‐DBS to the treatment of TRD (*n* = 1) and BD (*n* = 6), three BD patients exhibited substantial symptomatic remission 12 months after the intervention, which corresponded to a 100% improvement rate (C. Zhang et al. 2022). In contrast, our patient achieved a 47.06% improvement rate at the 12‐month follow‐up and an 88.24% improvement at the 24‐month follow‐up on the Bipolar Mania Rating Scale, which parallels the favorable outcomes reported in previous studies. Taken together, these observations underscore the efficacy of LHb‐DBS in mitigating the manic symptoms of BD. The choice of an invasive procedure like DBS over noninvasive techniques such as rTMS warrants discussion. While rTMS is a valuable, well‐tolerated treatment for depressive episodes in BD, its efficacy in the most severe, chronic, and multi‐episodic forms of TRBD can be limited and often transient (Nguyen et al. 2021). In this case, the patient had exhausted multiple pharmacological regimens, psychotherapy, and a trial of rTMS without sustained benefit, rendering her condition profoundly treatment‐resistant. The rationale for opting for LHb‐DBS was based on its potential for continuous, high‐precision modulation of a deep brain circuit implicated in the pathophysiology of BD, which is not directly accessible with noninvasive methods (C. Zhang et al. 2022; Sartorius and Henn 2007). The habenula is a key node in the reward and monoamine systems, and its dysregulation is strongly linked to depressive phenotypes, making it a rationally compelling target for neuromodulation (Abraham et al. 2023; Germann et al. 2021). This potential for sustained efficacy must be balanced against the inherent risks of neurosurgery (e.g., hemorrhage and infection) and hardware‐related complications (Rabins et al. 2009). For this patient, after extensive multidisciplinary evaluation and informed consent, the potential benefits of achieving long‐term stability and functional recovery were deemed to outweigh the risks.

### 4.3 Changes in fALFF Following LHb‐DBS

Previous research has consistently highlighted that abnormalities in the reward processing circuity are a core feature of BD pathophysiology (Johnson et al. 2012; Alloy et al. 2015). In addition, emerging evidence has suggested that individuals at risk for BD exhibit abnormalities in reward‐related brain function (Nusslock et al. 2014). Therefore, we focused specifically on regions within the reward circuit.

The fALFF score is a sensitive indicator of resting‐state brain activity (K. Zhang et al. 2017). Previous studies have reported abnormal functional activation in the limbic system of BD patients (Townsend and Altshuler 2012; Ahmed et al. 2023). Recent research using LHb‐DBS in rat models of depression has highlighted the crucial role of limbic and monoaminergic system activation in the induction of rapid antidepressant effects (Li et al. 2023). Monoaminergic systems, involving dopamine (DA), serotonin (5‐HT), noradrenaline (NA), and histamine, are involved in virtually all cerebral functions (Di Giovanni et al. 2016). These systems include dopaminergic neurons in the VTA and SNc (Di Giovanni et al. 2016). A core feature of BD is the vulnerability toward switching between low (depression) and high activity (mania) (Young and Dulcis 2015). The midbrain DA cell system, which consists of two major groups of projection cells in the substantia nigra and VTA (Grison et al. 2014), may exhibit instability in activity regulation (Loonen et al. 2017). A mouse model of mania showed that normalizing DA activity in the VTA can reverse anxiety‐related behaviors (Coque et al. 2011). Although few studies have focused specifically on the SNc in BD, normalization of SNc activity, an area predominantly composed of densely packed dopaminergic neurons (Carmichael et al. 2021), may be associated with symptom relief. The putamen, which is part of the basal nuclei involved in limbic system functions (Lanciego et al. 2012), is less active in BD patients than in controls (Chen et al. 2011). Our results reveal that chronic LHb‐DBS increases the activation of key regions such as the putamen, SNc, and VTA. This suggests that high‐frequency LHb‐DBS in BD patients enhances brain activity within the reward system, and normalizes activity in the putamen, VTA, and SNc, which leads to symptom improvement.

### 4.4 Changes in rsFC Following LHb‐DBS

The NAcc plays a central role within the reward network (Whittaker et al. 2018), and its intrinsic connectivity with the hippocampus is well‐documented (Lisman et al. 2011; Shohamy and Adcock 2010). Previous studies have linked heightened structural connectivity of the NAcc with a propensity for hypomania, which suggests that increased connectivity of the NAcc predict manic tendencies in BD (Damme et al. 2017). We found a consistent decrease in rsFC between the left NAcc and the left hippocampus following stimulation. This decline may indicate mitigation of excessive activation after 15 months of stimulation. The reduced connectivity may have contributed to the improvement manic symptoms. Specifically, the changes in fALFF and rsFC from baseline with DBS off to 15 months may reflect the neuroplastic changes due to 15 months of LHb‐DBS.

The application of LHb‐DBS to the treatment of TRBD represents a novel therapeutic approach with numerous advantages. First, this technique is recognized for its inherent safety and reversibility, which enables the modulation of patient symptomatology via fine adjustments of stimulation parameters. Such refinements enable the effective management of mood fluctuations and the concomitant enhancement of quality of life. Second, LHb‐DBS induces minimal adverse effects in patients, while avoiding the side effects of pharmacological interventions. Finally, LHb‐DBS offers a superior long‐term prognosis, with the potential to significantly elevate the quality of life of individuals with TRBD.

## 5 Limitations

Our research has certain limitations. First, the single‐case design and the lack of a control experiment limit the generalizability of our findings and may render our results susceptible to bias. Second, the patient remained on a stable regimen of mood stabilizers throughout the study. While this consistency allows for observing the added effect of DBS, it remains a potential confound, making it challenging to absolutely isolate the effects of DBS from those of concurrent pharmacotherapy. Future studies with larger samples and designed medication tapers would be valuable to address this. We aim to improve and refine our experimental design in future studies.

## 6 Conclusions

In our study, we found that long‐term LHb‐DBS can control symptoms and improve the quality of life of patients with TRBD. The mechanism underlying the therapeutic effect may be an increase in fALFF in the putamen, VTA, and SNc, and a reduction in rsFC between the left NAcc and left hippocampus.

## Author Contributions

**Chao Jiang**: formal analysis, methodology, project administration, resources, writing – original draft. **Peijing Du**: formal analysis, investigation, methodology, resources, writing – original draft. **Lingxiao Guan**: investigation, methodology, resources. **Chunhua Hu**: data curation, funding acquisition, investigation, project administration, supervision, validation. **Zhiyan Wang**: data curation, funding acquisition. **Tengteng Fan**: investigation, methodology, resources. **Jian Wang**: investigation, methodology, resources. **Zhiqiang Cui**: conceptualization, data curation, funding acquisition, investigation, methodology, resources, supervision, validation, writing – review and editing. All authors have read and approved the final submitted version of the manuscript.

## Ethics Statement

The studies involving human participants were reviewed and approved by Chinese People's Liberation Army General Hospital Medical Ethics Committee. The patients/participants provided their written informed consent to participate in this study. Written informed consent was obtained from the individual(s) for the publication of any potentially identifiable images or data included in this article.

## Conflicts of Interest

The authors declare no conflicts of interest.

## Peer Review

The peer review history for this article is available at https://publons.com/publon/10.1002/brb3.70899.

## Data Availability

The original contributions presented in the study are included in the article/Supporting Information, further inquiries can be directed to the corresponding authors.

## References

[brb370899-bib-0001] Abraham, M. E. , V. Ong , J. Gendreau , et al. 2023. “Investigating Deep Brain Stimulation of the Habenula: A Review of Clinical Studies.” Neuromodulation 26, no. 2: 292–301. 35840520 10.1016/j.neurom.2022.05.005

[brb370899-bib-0002] Ahmed, Y. B. , A. N. Al‐Bzour , S. M. Alzghoul , et al. 2023. “Limbic and Cortical Regions as Functional Biomarkers Associated With Emotion Regulation in Bipolar Disorder: A Meta‐Analysis of Neuroimaging Studies.” Journal of Affective Disorders 323: 506–513. 36462610 10.1016/j.jad.2022.11.071

[brb370899-bib-0003] Alloy, L. B. , R. Nusslock , and E. M. Boland . 2015. “The Development and Course of Bipolar Spectrum Disorders: An Integrated Reward and Circadian Rhythm Dysregulation Model.” Annual Review of Clinical Psychology 11: 213–250. 10.1146/annurev-clinpsy-032814-112902 PMC4380533 25581235

[brb370899-bib-0004] Bech, P 2002. “The Bech‐Rafaelsen Melancholia Scale (MES) in Clinical Trials of Therapies in Depressive Disorders: A 20‐Year Review of Its Use as Outcome Measure.” Acta Psychiatrica Scandinavica 106, no. 4: 252–264. 12225492 10.1034/j.1600-0447.2002.01404.x

[brb370899-bib-0005] Berk, M. , S. Dodd , M. Kauer‐Sant'anna , et al. 2007. “Dopamine Dysregulation Syndrome: Implications for a Dopamine Hypothesis of Bipolar Disorder.” Acta Psychiatrica Scandinavica Supplementum 116, no. 434: 41–49. 10.1111/j.1600-0447.2007.01058.x 17688462

[brb370899-bib-0006] Buysse, D. J. , C. F. Reynolds 3rd , T. H. Monk , S. R. Berman , and D. J. Kupfer . 1989. “The Pittsburgh Sleep Quality Index: A New Instrument for Psychiatric Practice and Research.” Psychiatry Research 28, no. 2: 193–213. 2748771 10.1016/0165-1781(89)90047-4

[brb370899-bib-0007] Carmichael, K. , B. Sullivan , E. Lopez , L. Sun , and H. Cai . 2021. “Diverse Midbrain Dopaminergic Neuron Subtypes and Implications for Complex Clinical Symptoms of Parkinson's Disease.” Ageing and Neurodegenerative Diseases 1, no. 4: 10–20517. 10.20517/and.2021.07 PMC8442626 34532720

[brb370899-bib-0008] Cha, J. , K. S. Choi , J. K. Rajendra , et al. 2024. “Whole Brain Network Effects of Subcallosal Cingulate Deep Brain Stimulation for Treatment‐Resistant Depression.” Molecular Psychiatry 29, no. 1: 112–120. 37919403 10.1038/s41380-023-02306-6 PMC11078711

[brb370899-bib-0009] Chen, C. H. , J. Suckling , B. R. Lennox , C. Ooi , and E. T. Bullmore . 2011. “A Quantitative Meta‐Analysis of fMRI Studies in Bipolar Disorder.” Bipolar Disorders 13, no. 1: 1–15. 10.1111/j.1399-5618.2011.00893.x 21320248

[brb370899-bib-0010] Coque, L. , S. Mukherjee , J. L. Cao , et al. 2011. “Specific Role of VTA Dopamine Neuronal Firing Rates and Morphology in the Reversal of Anxiety‐Related, but Not Depression‐Related Behavior in the ClockDelta19 Mouse Model of Mania.” Neuropsychopharmacology 36, no. 7: 1478–1488. 21430648 10.1038/npp.2011.33 PMC3096816

[brb370899-bib-0011] Cui, Z. , C. Jiang , C. Hu , et al. 2023. “Safety and Precision of Frontal Trajectory of Lateral Habenula Deep Brain Stimulation Surgery in Treatment‐Resistant Depression.” Frontiers in Neurology 14: 1113545. 37006495 10.3389/fneur.2023.1113545 PMC10060811

[brb370899-bib-0012] Damme, K. S. , C. B. Young , and R. Nusslock . 2017. “Elevated Nucleus Accumbens Structural Connectivity Associated With Proneness to Hypomania: A Reward Hypersensitivity Perspective.” Social Cognitive and Affective Neuroscience 12, no. 6: 928–936. 28338785 10.1093/scan/nsx017 PMC5472153

[brb370899-bib-0013] Di Giovanni, G. , D. Svob Strac , M. Sole , et al. 2016. “Monoaminergic and Histaminergic Strategies and Treatments in Brain Diseases.” Frontiers in Neuroscience 10: 541. 27932945 10.3389/fnins.2016.00541 PMC5121249

[brb370899-bib-0014] Fountoulakis, K. N. , H. Grunze , E. Vieta , et al. 2017. “The International College of Neuro‐Psychopharmacology (CINP) Treatment Guidelines for Bipolar Disorder in Adults (CINP‐BD‐2017), Part 3: The Clinical Guidelines.” International Journal of Neuropsychopharmacology 20, no. 2: 180–195. 27941079 10.1093/ijnp/pyw109 PMC5408976

[brb370899-bib-0015] Friston, K. J. , S. Williams , R. Howard , R. S. Frackowiak , and R. Turner . 1996. “Movement‐Related Effects in fMRI Time‐Series.” Magnetic Resonance in Medicine 35, no. 3: 346–355. 8699946 10.1002/mrm.1910350312

[brb370899-bib-0016] Germann, J. , M. Mameli , G. J. B. Elias , et al. 2021. “Deep Brain Stimulation of the Habenula: Systematic Review of the Literature and Clinical Trial Registries.” Frontiers in Psychiatry 12: 730931. 34484011 10.3389/fpsyt.2021.730931 PMC8415908

[brb370899-bib-0017] Graat, I. , G. van Rooijen , R. Mocking , et al. 2020. “Is Deep Brain Stimulation Effective and Safe for Patients With Obsessive Compulsive Disorder and Comorbid Bipolar Disorder?” Journal of Affective Disorders 264: 69–75. 31846903 10.1016/j.jad.2019.11.152

[brb370899-bib-0018] Grison, A. , S. Zucchelli , A. Urzi , et al. 2014. “Mesencephalic Dopaminergic Neurons Express a Repertoire of Olfactory Receptors and Respond to Odorant‐Like Molecules.” BMC Genomics 15, no. 1: 729. 25164183 10.1186/1471-2164-15-729 PMC4161876

[brb370899-bib-0019] Hamilton, M 1959. “The Assessment of Anxiety States by Rating.” British Journal of Medical Psychology 32, no. 1: 50–55. 13638508 10.1111/j.2044-8341.1959.tb00467.x

[brb370899-bib-0020] Hamilton, M 1960. “A Rating Scale for Depression.” Journal of Neurology, Neurosurgery, and Psychiatry 23, no. 1: 56–62. 14399272 10.1136/jnnp.23.1.56 PMC495331

[brb370899-bib-0021] Hikosaka, O 2010. “The Habenula: From Stress Evasion to Value‐Based Decision‐Making.” Nature Reviews Neuroscience 11, no. 7: 503–513. 20559337 10.1038/nrn2866 PMC3447364

[brb370899-bib-0022] Holtzheimer, P. E. , M. E. Kelley , R. E. Gross , et al. 2012. “Subcallosal Cingulate Deep Brain Stimulation for Treatment‐Resistant Unipolar and Bipolar Depression.” Archives of General Psychiatry 69, no. 2: 150–158. 22213770 10.1001/archgenpsychiatry.2011.1456 PMC4423545

[brb370899-bib-0023] Hu, H. , Y. Cui , and Y. Yang . 2020. “Circuits and Functions of the Lateral Habenula in Health and in Disease.” Nature Reviews Neuroscience 21, no. 5: 277–295. 32269316 10.1038/s41583-020-0292-4

[brb370899-bib-0024] Hwang, J. , I. K. Lyoo , S. R. Dager , et al. 2006. “Basal Ganglia Shape Alterations in Bipolar Disorder.” American Journal of Psychiatry 163, no. 2: 276–285. 16449482 10.1176/appi.ajp.163.2.276

[brb370899-bib-0025] Johnson, S. L. , M. D. Edge , M. K. Holmes , and C. S. Carver . 2012. “The Behavioral Activation System and Mania.” Annual Review of Clinical Psychology 8: 243–267. 10.1146/annurev-clinpsy-032511-143148 PMC3409638 22077912

[brb370899-bib-0026] Lanciego, J. L. , N. Luquin , and J. A. Obeso . 2012. “Functional Neuroanatomy of the Basal Ganglia.” Cold Spring Harbor Perspectives in Medicine 2, no. 12: a009621. 23071379 10.1101/cshperspect.a009621 PMC3543080

[brb370899-bib-0027] Li, G. , B. Bo , P. Wang , et al. 2023. “Instantaneous Antidepressant Effect of Lateral Habenula Deep Brain Stimulation in Rats Studied With Functional MRI.” Elife 12: e84693. 37261976 10.7554/eLife.84693 PMC10234627

[brb370899-bib-0028] Lisman, J. , A. A. Grace , and E. Duzel . 2011. “A neoHebbian Framework for Episodic Memory; Role of Dopamine‐Dependent Late LTP.” Trends in Neuroscience 34, no. 10: 536–547. 10.1016/j.tins.2011.07.006 PMC3183413 21851992

[brb370899-bib-0029] Loonen, A. J. M. , R. W. Kupka , and S. A. Ivanova . 2017. “Circuits Regulating Pleasure and Happiness in Bipolar Disorder.” Frontiers in Neural Circuits 11: 35. 28588455 10.3389/fncir.2017.00035 PMC5439000

[brb370899-bib-0030] Malone, D. A., Jr. , D. D. Dougherty , A. R. Rezai , et al. 2009. “Deep Brain Stimulation of the Ventral Capsule/Ventral Striatum for Treatment‐Resistant Depression.” Biological Psychiatry 65, no. 4: 267–275. 18842257 10.1016/j.biopsych.2008.08.029 PMC3486635

[brb370899-bib-0031] Matsumoto, M. , and O. Hikosaka . 2007. “Lateral Habenula as a Source of Negative Reward Signals in Dopamine Neurons.” Nature 447, no. 7148: 1111–1115. 17522629 10.1038/nature05860

[brb370899-bib-0032] McIntyre, R. S. , M. Berk , E. Brietzke , et al. 2020. “Bipolar Disorders.” Lancet 396, no. 10265: 1841–1856. 33278937 10.1016/S0140-6736(20)31544-0

[brb370899-bib-0033] Montgomery, S. A. , and M. Asberg . 1979. “A New Depression Scale Designed to be Sensitive to Change.” British Journal of Psychiatry 134: 382–389. 10.1192/bjp.134.4.382 444788

[brb370899-bib-0034] Mutz, J 2023. “Brain Stimulation Treatment for Bipolar Disorder.” Bipolar Disorders 25, no. 1: 9–24. 10.1111/bdi.13283 PMC10210071 36515461

[brb370899-bib-0035] Nguyen, T. D. , F. Hieronymus , R. Lorentzen , A. McGirr , and S. D. Ostergaard . 2021. “The Efficacy of Repetitive Transcranial Magnetic Stimulation (rTMS) for Bipolar Depression: A Systematic Review and Meta‐Analysis.” Journal of Affective Disorders 279: 250–255. 33074144 10.1016/j.jad.2020.10.013

[brb370899-bib-0036] Nierenberg, A. A. , B. Agustini , O. Kohler‐Forsberg , et al. 2023. “Diagnosis and Treatment of Bipolar Disorder: A Review.” JAMA 330, no. 14: 1370–1380. 37815563 10.1001/jama.2023.18588

[brb370899-bib-0037] Nusslock, R. , and L. B. Alloy . 2017. “Reward Processing and Mood‐Related Symptoms: An RDoC and Translational Neuroscience Perspective.” Journal of Affective Disorders 216: 3–16. 28237133 10.1016/j.jad.2017.02.001 PMC6661152

[brb370899-bib-0038] Nusslock, R. , C. B. Young , and K. S. Damme . 2014. “Elevated Reward‐Related Neural Activation as a Unique Biological Marker of Bipolar Disorder: Assessment and Treatment Implications.” Behaviour Research and Therapy 62: 74–87. 25241675 10.1016/j.brat.2014.08.011 PMC6727647

[brb370899-bib-0039] Rabins, P. , B. S. Appleby , J. Brandt , et al. 2009. “Scientific and Ethical Issues Related to Deep Brain Stimulation for Disorders of Mood, Behavior, and Thought.” Archives of General Psychiatry 66, no. 9: 931–937. 19736349 10.1001/archgenpsychiatry.2009.113 PMC2753479

[brb370899-bib-0040] Rush, A. J. , M. H. Trivedi , H. M. Ibrahim , et al. 2003. “The 16‐Item Quick Inventory of Depressive Symptomatology (QIDS), Clinician Rating (QIDS‐C), and Self‐Report (QIDS‐SR): A Psychometric Evaluation in Patients With Chronic Major Depression.” Biological Psychiatry 54, no. 5: 573–583. 12946886 10.1016/s0006-3223(02)01866-8

[brb370899-bib-0041] Sachs, G. S. 1996. “Treatment‐Resistant Bipolar Depression.” Psychiatric Clinics of North America 19, no. 2: 215–236. 8827187 10.1016/s0193-953x(05)70285-9

[brb370899-bib-0042] Sachs, G. S. , J. M. Dupuy , and C. W. Wittmann . 2011. “The Pharmacologic Treatment of Bipolar Disorder.” Journal of Clinical Psychiatry 72, no. 5: 704–715. 21658351 10.4088/JCP.10m06523

[brb370899-bib-0043] Sartorius, A. , and F. A. Henn . 2007. “Deep Brain Stimulation of the Lateral Habenula in Treatment Resistant Major Depression.” Medical Hypotheses 69, no. 6: 1305–1308. 17498883 10.1016/j.mehy.2007.03.021

[brb370899-bib-0044] Schlaepfer, T. E. , B. H. Bewernick , S. Kayser , B. Madler , and V. A. Coenen . 2013. “Rapid Effects of Deep Brain Stimulation for Treatment‐Resistant Major Depression.” Biological Psychiatry 73, no. 12: 1204–1212. 23562618 10.1016/j.biopsych.2013.01.034

[brb370899-bib-0045] Schulder, M. , A. Mishra , A. Mammis , et al. 2023. “Advances in Technical Aspects of Deep Brain Stimulation Surgery.” Stereotactic and Functional Neurosurgery 101, no. 2: 112–134. 36809747 10.1159/000529040 PMC10184879

[brb370899-bib-0046] Schultz, W 2016. “Dopamine Reward Prediction Error Coding.” Dialogues in Clinical Neuroscience 18, no. 1: 23–32. 27069377 10.31887/DCNS.2016.18.1/wschultz PMC4826767

[brb370899-bib-0047] Shohamy, D. , and R. A. Adcock . 2010. “Dopamine and Adaptive Memory.” Trends in Cognitive Sciences 14, no. 10: 464–472. 20829095 10.1016/j.tics.2010.08.002

[brb370899-bib-0048] Stamatakis, A. M. , J. H. Jennings , R. L. Ung , et al. 2013. “A Unique Population of Ventral Tegmental Area Neurons Inhibits the Lateral Habenula to Promote Reward.” Neuron 80, no. 4: 1039–1053. 24267654 10.1016/j.neuron.2013.08.023 PMC3873746

[brb370899-bib-0049] Strakowski, S. M. , C. M. Adler , S. K. Holland , N. P. Mills , M. P. DelBello , and J. C. Eliassen . 2005. “Abnormal FMRI Brain Activation in Euthymic Bipolar Disorder Patients During a Counting Stroop Interference Task.” American Journal of Psychiatry 162, no. 9: 1697–1705. 16135630 10.1176/appi.ajp.162.9.1697

[brb370899-bib-0050] Strakowski, S. M. , M. P. DelBello , M. E. Zimmerman , et al. 2002. “Ventricular and Periventricular Structural Volumes in First‐ Versus Multiple‐Episode Bipolar Disorder.” American Journal of Psychiatry 159, no. 11: 1841–1847. 12411217 10.1176/appi.ajp.159.11.1841

[brb370899-bib-0051] Svenaeus, F 2014. “Diagnosing Mental Disorders and Saving the Normal: American Psychiatric Association, 2013. Diagnostic and Statistical Manual of Mental Disorders, 5th ed. American Psychiatric Publishing. 991 pp., ISBN: 978–0890425558.” Medicine, Health Care, and Philosophy 17, no. 2: 241–244.

[brb370899-bib-0052] Swartz, H. A. , and J. Swanson . 2014. “Psychotherapy for Bipolar Disorder in Adults: A Review of the Evidence.” Focus 12, no. 3: 251–266. 26279641 10.1176/appi.focus.12.3.251 PMC4536930

[brb370899-bib-0053] Tondo, L. , G. H. Vazquez , and R. J. Baldessarini . 2017. “Depression and Mania in Bipolar Disorder.” Current Neuropharmacology 15, no. 3: 353–358. 28503106 10.2174/1570159X14666160606210811 PMC5405618

[brb370899-bib-0054] Torres, C. V. , E. Ezquiaga , M. Navas , M. A. Garcia Pallero , and R. G. Sola . 2017. “Long‐Term Results of Deep Brain Stimulation of the Subcallosal Cingulate for Medication‐Resistant Bipolar I Depression and Rapid Cycling Bipolar II Depression.” Biological Psychiatry 81, no. 4: e33–e34. 27524499 10.1016/j.biopsych.2016.05.026

[brb370899-bib-0055] Townsend, J. , and L. L. Altshuler . 2012. “Emotion Processing and Regulation in Bipolar Disorder: A Review.” Bipolar Disorders 14, no. 4: 326–339. 22631618 10.1111/j.1399-5618.2012.01021.x

[brb370899-bib-0056] Whitfield‐Gabrieli, S. , and A. Nieto‐Castanon . 2012. “Conn: A Functional Connectivity Toolbox for Correlated and Anticorrelated Brain Networks.” Brain Connectivity 2, no. 3: 125–141. 22642651 10.1089/brain.2012.0073

[brb370899-bib-0057] Whittaker, J. R. , S. F. Foley , E. Ackling , K. Murphy , and X. Caseras . 2018. “The Functional Connectivity Between the Nucleus Accumbens and the Ventromedial Prefrontal Cortex as an Endophenotype for Bipolar Disorder.” Biological Psychiatry 84, no. 11: 803–809. 30227973 10.1016/j.biopsych.2018.07.023 PMC6218647

[brb370899-bib-0058] Xia, M. , J. Wang , and Y. He . 2013. “BrainNet Viewer: A Network Visualization Tool for Human Brain Connectomics.” PLoS ONE 8, no. 7: e68910. 23861951 10.1371/journal.pone.0068910 PMC3701683

[brb370899-bib-0059] Yan, C. G. , X. D. Wang , X. N. Zuo , and Y. F. Zang . 2016. “DPABI: Data Processing & Analysis for (Resting‐State) Brain Imaging.” Neuroinformatics 14, no. 3: 339–351. 27075850 10.1007/s12021-016-9299-4

[brb370899-bib-0060] Yang, Y. , H. Wang , J. Hu , and H. Hu . 2018. “Lateral Habenula in the Pathophysiology of Depression.” Current Opinion in Neurobiology 48: 90–96. 29175713 10.1016/j.conb.2017.10.024

[brb370899-bib-0061] Young, J. W. , and D. Dulcis . 2015. “Investigating the Mechanism(s) Underlying Switching Between States in Bipolar Disorder.” European Journal of Pharmacology 759: 151–162. 25814263 10.1016/j.ejphar.2015.03.019 PMC4437855

[brb370899-bib-0062] Zhang, C. , S. G. Kim , D. Li , et al. 2019. “Habenula Deep Brain Stimulation for Refractory Bipolar Disorder.” Brain Stimulation: Basic, Translational, and Clinical Research in Neuromodulation 12, no. 5: 1298–1300. 10.1016/j.brs.2019.05.010 31103455

[brb370899-bib-0063] Zhang, C. , Y. Zhang , H. Luo , et al. 2022. “Bilateral Habenula Deep Brain Stimulation for Treatment‐Resistant Depression: Clinical Findings and Electrophysiological Features.” Translational Psychiatry 12, no. 1: 52. 35115488 10.1038/s41398-022-01818-z PMC8813927

[brb370899-bib-0064] Zhang, K. , Z. Liu , X. Cao , et al. 2017. “Amplitude of Low‐Frequency Fluctuations in First‐Episode, Drug‐Naive Depressive Patients: A 5‐Year Retrospective Study.” PLoS ONE 12, no. 4: e0174564. 28384269 10.1371/journal.pone.0174564 PMC5383053

